# Simulation-based Remediation in Emergency Medicine Residency Training: A Consensus Study

**DOI:** 10.5811/westjem.2018.10.39781

**Published:** 2018-11-20

**Authors:** Nur-Ain Nadir, Danielle Hart, Michael Cassara, Joan Noelker, Tiffany Moadel, Miriam Kulkarni, Christopher S. Sampson, Suzanne Bentley, Neel K. Naik, Jessica Hernandez, Sara M. Krzyzaniak, Steven Lai, Gregory Podolej, Christopher Strother

**Affiliations:** * Kaiser Permanente Central Valley, Department of Emergency Medicine, Modesto, California; † University of Minnesota, Hennepin County Medical Center, Department of Emergency Medicine, Minneapolis, Minnesota; ‡ Donald and Barbara Zucker School of Medicine at Hofstra-Northwell, Department of Emergency Medicine, Manhasset, New York; § Washington University in St. Louis, Department of Medicine, Division of Emergency Medicine, St. Louis, Missouri; ¶ St. John’s Riverside Hospital, Department of Emergency Medicine, Yonkers, New York; || University of Missouri-Columbia, Department of Emergency Medicine, Columbia, Missouri; # Icahn School of Medicine at Mount Sinai, Elmhurst Hospital Center, Department of Emergency Medicine and Medical Education, Simulation Center, Elmhurst, New York; ** New York Presbyterian, Weill Cornell Medicine, Department of Emergency Medicine, New York, New York; †† University of Texas Southwestern Medical Center, Department of Emergency Medicine, Dallas, Texas; ‡‡ Ronald Reagan UCLA Medical Center, Olive View-UCLA Medical Center, Department of Emergency Medicine, Los Angeles, California; §§ Icahn School of Medicine at Mount Sinai, Department of Emergency Medicine, Pediatrics, and Medical Education, New York, New York; ¶¶ University of Illinois-Peoria, Jump Simulation, Peoria, Illinois

## Abstract

**Introduction:**

Resident remediation is a pressing topic in emergency medicine (EM) training programs. Simulation has become a prominent educational tool in EM training and been recommended for identification of learning gaps and resident remediation. Despite the ubiquitous need for formalized remediation, there is a dearth of literature regarding best practices for simulation-based remediation (SBR).

**Methods:**

We conducted a literature search on SBR practices using the terms “simulation,” “remediation,” and “simulation based remediation.” We identified relevant themes and used them to develop an open-ended questionnaire that was distributed to EM programs with experience in SBR. Thematic analysis was performed on all subsequent responses and used to develop survey instruments, which were then used in a modified two-round Delphi panel to derive a set of consensus statements on the use of SBR from an aggregate of 41 experts in simulation and remediation in EM.

**Results:**

Faculty representing 30 programs across North America composed the consensus group with 66% of participants identifying themselves as simulation faculty, 32% as program directors, and 2% as core faculty. The results from our study highlight a strong agreement across many areas of SBR in EM training. SBR is appropriate for a range of deficits, including procedural, medical knowledge application, clinical reasoning/decision-making, communication, teamwork, and crisis resource management. Simulation can be used both diagnostically and therapeutically in remediation, although SBR should be part of a larger remediation plan constructed by the residency leadership team or a faculty expert in remediation, and not the only component. Although summative assessment can have a role in SBR, it needs to be very clearly delineated and transparent to everyone involved.

**Conclusion:**

Simulation may be used for remediation purposes for certain specific kinds of competencies as long as it is carried out in a transparent manner to all those involved.

## INTRODUCTION

With the Accreditation Council of Graduate Medical Education’s (ACGME) adoption of a competency-based (CB) educational framework, and the majority of emergency medicine (EM) residencies reporting at least one resident on probationary status, remediation has become a pressing topic in EM resident education.^1^,^2^ Some residency training programs struggle with the paradox between the foundational premise of CB training (i.e., a time-independent path to competence for all learners) and the ACGME’s prescribed length of residency training (three or four years for EM residency training).^3^,^4^ The fact that not all learners achieve competence at the same time or rates^2^ further compounds the matter, necessitating remediation plans for learners falling outside the competency bell curve.^5^,^6^

“Remediation” can be used to describe the status of a resident within a program (such as “probation”) or the “effort spent to improve a resident’s knowledge, skills, or attitudes.”^7^ In this project, remediation is defined as any additional training, instruction, or practice provided to residents found to be deficient in one of the six core competencies in EM.^5^,^7^ Note that remediation is not necessarily equivalent to probation, which implies a formal notation on a resident’s academic file; remediation may occur informally without an annotation or the resident being formally under probation.^5^

The last two decades have seen an increase in the use of simulation pedagogies, such as simulation-based mastery learning (SBML) in EM resident education,^8^–^11^ and there are reports of SBML successfully being used for procedural education.^9^,^12^ SBML lends itself particularly to the CB approach in that it is time-independent, allowing learners to achieve mastery over time.^9^,^10^,^13^ There are also anecdotal reports of success with other kinds of simulation models in EM. Simulation modalities such as high-fidelity patient simulators (mannequins), standardized patients, partial task trainers, computer screen-based simulation, virtual reality environments, and tabletop role-playing exercises such as oral board exam-style simulations have been used to create opportunities and safe environments for clinical training^14^ and anecdotally for remediation. While over 90% of EM residency programs use some form of simulation,^11^,^15^ how exactly it is being used and the principles guiding its use vary widely. Current recommendations, in general, support the incorporation of simulation into curricula for instruction, identification of knowledge gaps, evaluation and remediation.^8^,^16^–^24^

The successful use of simulation-based remediation (SBR) in other specialties and fields such as anesthesia, internal medicine, and nursing have been described, but the concept in general is under-reported.^17^,^18^,^20^,^25^–^27^ Evidence supporting the use of SBR within EM training is somewhat contradictory. In 2007 a Society of Academic Emergency Medicine (SAEM) task force on simulation research in EM cautioned against the use of SBR, contending that the term “remediation” could not be reliably applied, given differences in faculty perception of resident performance.^28^ In 2012, the ACGME published 23 EM sub-competencies to be used in the assessment of EM residents. They suggest using simulation as one method to evaluate sub-competencies 1–11 and 16–23.^29^ Some authorities have posited that since simulation could be used for specific sub-competency assessment, it could also be used for remediation within those same sub-competencies.^17^,^18^ In 2016, the Council Of Residency Directors (CORD)-EM Remediation Task Force (RTF) recommended simulation for remediating multiple competencies, including patient-centered communication, teamwork and leadership.^16^,^30^

**Population Health Research Capsule**

What do we already know about this issue?

*With the Accreditation Council of Graduate Medical Education’s transition towards a competency-based framework, simulation-based remediation (SBR) has become a pressing topic; however, few guidelines exist to direct its use*.

What was the research question?

*Authors sought to develop consensus on the appropriate use of SBR in emergency medicine residency training programs*.

What was the major finding of the study?

*SBR can be used for remediating specific competencies provided there is process and outcome transparency*.

How does this improve population health?

*SBR can assist in remediating learners so as to produce clinically competent physicians, thereby promoting patient safety and quality of care*.

Interestingly, many of the recommendations on the use of SBR arose from experts in EM residency leadership and remediation. Simulationists were possibly under-represented among the stakeholders making the aforementioned recommendations. This is relevant because some simulationists view the experience as formative and eschew the use of simulation for remediation purposes, arguing that remediation implies summative assessment, which intrinsically threatens the principle of “learner safety” integral to simulation-based education.^31^–^34^ There are also concerns that simulation for high-stakes assessment requires consensus on case design standards and setting of minimum performance levels to ensure that the testing is valid.^35^

With the exception of SBML and procedural remediation,^9^ there remains no clear consensus on when and how to appropriately use simulation for remediation in EM for other sub-competencies. The lack of specific recommendations and guidelines makes SBR planning difficult for both residency program and simulation leadership. To answer this need, the CORD-EM Simulation Community of Practice (COP), CORD-EM RTF and the SAEM Simulation Academy formed a joint collaboration, the Simulation Based Remediation Collaborative (SBRC), to clarify the role of SBR. This study was based on their work, and its objective was to build consensus on the appropriate use of SBR in EM.

## METHODS

**Figure 1:**
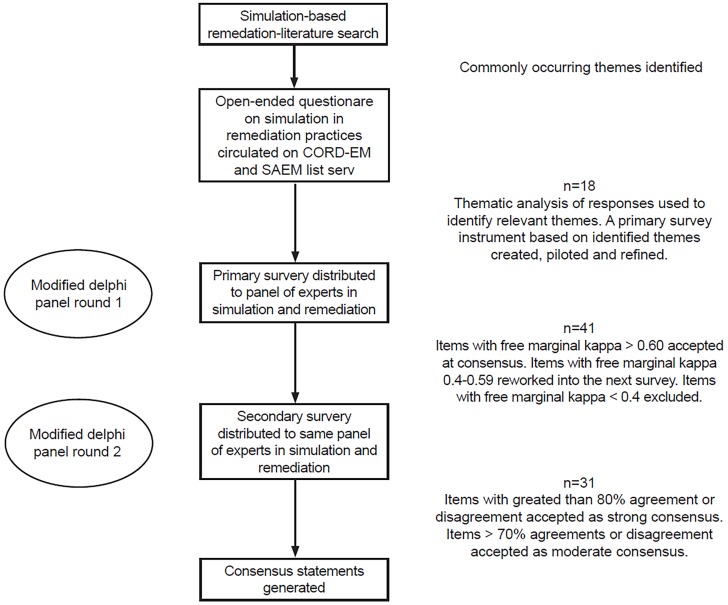
Study design. *CORD-EM*, Council of Emergency Medicine Residency Directors; *SAEM*, Society for Academic Emergency Medicine.

This study was deemed exempt by the local institutional review board. Using previously described methodology, we employed a modified Delphi approach to derive a set of consensus statements on using simulation for remediation in EM.^36^–^40^ The study design is depicted in Figure 1. We conducted a literature search on simulation remediation practices using terms “simulation,” “remediation,” and “simulation based remediation,” and identified commonly occurring themes. Using these themes, we created an open-ended questionnaire. From May through June 2017, the CORD-EM listserv was queried for all programs with experience in SBR. The questionnaire was subsequently sent to the 18 programs that indicated experience in SBR. Responses to the questionnaire were assessed using thematic analysis^41^ by two EM simulation remediation experts (NN, GP) and an EM remediation expert (SK). We used the commonly occurring themes to create an initial survey, which was then piloted among a group of six EM simulation and remediation experts (NN, CS, MK, DH, JN, TM) and further refined based on their input.

We circulated the final primary survey to 52 experts with experience using simulation for remediation purposes, who had been identified a priori through their involvement in the CORD-EM Simulation COP, the RTF, and SAEM Simulation Academy and any publications or presentations on SBR. Experts were requested to rank each statement according to the following categories: “agree,” “modify,” or “disagree;” and survey program parameters were set to completion of all survey items. The study group analyzed the results of the primary survey, and Randolph’s free marginal kappa was calculated to gauge agreement for each statement. Randolph’s free kappa is a chance-adjusted measure of agreement for any number of cases, categories, or raters^42^ and has been used to measure agreement in studies with large numbers of raters (experts).^39^,^40^

A free marginal kappa ≥ 0.6 was used to indicate good agreement.^42^,^43^ We removed items without consensus (free marginal kappa < 0.4). Items with moderate agreement (free marginal kappa 0.4–0.59) were reworked into a second survey. We also analyzed narrative comments from the initial survey and comments pertaining to “modifying” statements through thematic analysis, and any newly emerging themes were incorporated into the second survey. The second survey was distributed to the same panel of experts who had responded to the initial survey. On the secondary survey we used a cut off of 80% to indicate strong agreement and we deemed 70% moderate agreement with respect to consensus.

## RESULTS

**Figure 2:**
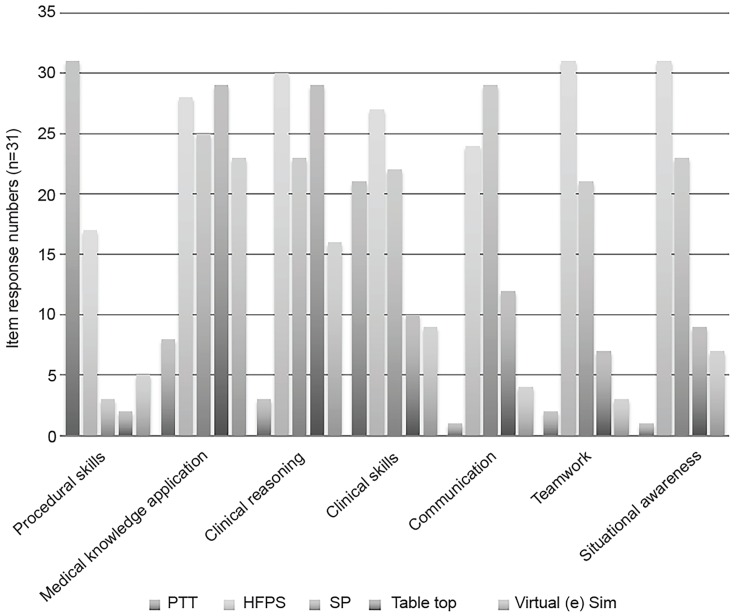
Simulation modalities best suited to specific deficiencies. *PTT*, partial task trainers; *HFPS*, high fidelity patient simulators (mannequins); *SP*, standardized patients. Tabletop: oral board-style simulations; Virtual (e) Sims Online web-based virtual simulations.

41 of 52 invited individuals completed Round 1 of the survey for a response rate of 78%, and 31 out of the initial 41 participants completed Round 2 for a response rate of 76%. Sixty-six percent of participants identified themselves primarily as simulation faculty, 32% identified themselves primarily as program directors (PDs) or assistant/associate program directors (APDs), and 2% identified themselves primarily as core faculty. Four of those identifying themselves as PDs/APDs also had simulation training. Experts represented 30 programs from across North America (Table 1). The modified Delphi process yielded 38 statements with strong agreement, eight with moderate agreement and nine with no agreement within six themes: 1) role of simulation in remediation; 2) decision to use simulation in remediation; 3) SBR process; 4) debriefing SBR; 5) assessing and reporting SBR; and 6) defining and determining SBR success. The modified Delphi process yielded 11 statements with strong agreement, one with moderate agreement and five with no agreement within the theme “deficiencies best addressed by SBR.” The modified Delphi process yielded 10 statements with strong agreement, two with moderate agreement and 11 with no agreement for “sub-competencies best addressed by SBR” (Table 2). Consensus in the alignment of simulation modalities to competency being remediated was also achieved (Figure 2).

## DISCUSSION

The results of our study show that there is strong agreement in many areas regarding SBR, including the belief that simulation can play a role in remediation. SBR should be part of a multifaceted remediation plan and not the *sole* remediation strategy. The residency leadership and the remediation faculty committee (or equivalent) should still be responsible for the overall remediation plan, with specific goals for the SBR components. These goals should be transparent to the learner and the faculty conducting the SBR. The methods used to assess the learner’s performance should be transparent and communicated to all stakeholders: the learner; the residency leadership; the remediation team; the clinical competency committee (CCC); and all other faculty involved in summative decisions regarding advancement. Although formative assessment is ideal, summative assessment may be employed, provided the process is clearly defined and transparent.

SBR may be used “diagnostically” and “therapeutically” to benefit the remediating learner. Diagnostic SBR provides a protected, standardized, and contextualized environment in which a learner’s performance gaps may be more precisely studied. In contrast to remediation where the struggling learner is situated within the clinical environment (under direct observation), the classroom, or an equivalent didactic setting, diagnostic SBR provides the conditions under which faculty and learners may safely and accurately explore the learners’ frames responsible for observed deviations from ideal performance. We posit that faculty are more likely to accurately identify the true reasons for performance gaps in the laboratory environment where SBR occurs (than in the clinical environment). While most faculty are likely able to directly observe their learners while contemporaneously working alongside them and identifying performance gaps, they are likely unable to learn *why* these gaps exist. In the challenging clinical milieu of today’s academic emergency department, where cognitively-loaded faculty and learners must balance the demands, expectations, and temporal pressures of patient care, there is no time, space, and privacy to support the reflection necessary for uncovering causes for performance gaps, which frequently tend to be multifactorial.^6^,^44^ As many residents struggle in multiple domains,^19^,^21^ diagnostic SBR may provide the best opportunity to identify one or more domains requiring attention. Diagnostic SBR, with a low resident-to-faculty ratio, may provide the best data to inform the development of an individualized remediation plan.

Modalities chosen for therapeutic SBR should be aligned with the learner’s needs. There is strong agreement that SBR is appropriate for areas such as application of medical knowledge, clinical reasoning, decision-making, communication, teamwork, leadership, crisis resource management (CRM), and cognitive overload/multitasking in high acuity situations. There is moderate agreement that SBR is not the best modality for developing foundational medical knowledge, as this may be best acquired through other means.

SBR seems most appropriate for the following sub-competencies: emergency stabilization, performance of a history and physical exam, diagnosis, pharmacotherapy, multitasking, and the procedural and communication milestones. Outside of CRM, simulation may not be the best modality for improving sub-competencies linked to the general competencies of systems-based practice (SBP) or problem-based learning and improvement (PBLI). While some aspects of PBLI could potentially be addressed in the debriefing portion of SBR (e.g., improving a learner’s insight through self-reflection following SBR, informing the development of an individualized development plan), this seems to be a small component of a larger PBLI remediation plan. While SBR for SBP may provide some opportunities for learners to practice mobilizing institutional or system resources to optimize patient care, the other aspects of this competency requiring remediation may necessitate the use of other strategies. Professionalism represents another domain more effectively addressed through means other than SBR. The Hawthorne effect could bias the assessment of a learner’s performance in a SBR conducted for professionalism concerns. While some learners may have difficulty with professionalism competencies in any circumstance, others may only display professionalism lapses when they are overly stressed, busy, frustrated, or not being directly observed.

The optimal number of SBR sessions required is difficult to define at the outset of a remediation plan and is dependent upon the focus of remediation and the learner’s progress. SBR focused on one domain requiring improvement has the potential to unmask another, which may necessitate a different simulation or non-simulation-based intervention. Learner improvement during each SBR session, therefore, informs the next steps to be taken. SBR for procedures incorporates the mastery learning approach,^9^,^10^,^45^,^46^ where the learner deliberately practices a procedure under facilitation until it is completed safely and competently in the simulated environment.^47^ For non-procedural SBR, learners should experience multiple simulation sessions of comparable cases with similar learning objectives in contrast to repeating the same exact simulation case (a practice that did not achieve agreement in our study) until those objectives are met.

Ideally, SBR would be conducted by faculty with formal simulation training or experience. However, only moderate agreement was obtained for the item “SBR may be conducted by the PD/APD (those ultimately involved in making progression decisions), as long as they have training in simulation.” One possible explanation for this moderate level of agreement is that residency leadership’s (PD/APD) direct involvement in SBR may be perceived as a threat to the principle of “learner safety.” While it may be optimal to have non-residency leadership faculty conduct SBR, we recognize the feasibility of this approach is dependent on the resources at the program; in some programs, the simulationist is part of the residency leadership. Programs should use the resources they have to optimize the learning and outcomes of SBR for their trainees.

With respect to assessment, our results, based on the final round of the Delphi panel, support the use of assessment tools with some validity evidence, similar to the work described by Blum et al.^25^ While procedural assessment tools with validity evidence exist,^48^,^49^ there are few simulation cases with validity evidence beyond content validity (i.e., internal structure, response process, relations to other variables, or consequential validity).^50^,^51^ Various assessment tools for non-technical skills also have been found to have some validity evidence, but without a co-existing recommended simulation case for EM resident learners. If assessment tools with validity evidence are available for the domain requiring remediation, these tools should be used to promote best practice. However, to maintain the validity of judgments made by using such an assessment tool, the tool must be applied to a similar population of learners, implemented under similar conditions, using similarly trained raters.

Tools with demonstrated validity evidence in one circumstance do not automatically demonstrate the same characteristics when applied to other circumstances. Unless validity evidence is re-demonstrated in the new contexts, there may not be current validity evidence for the tools chosen. Therefore, in agreement with previously published works by Blum et al.,^25^ we also recommend that summative (“high-stakes”) SBR should not be used as the *solitary* measure of a learner’s attainment of competency in a given domain. Summative SBR should be used as part of a comprehensive remediation plan providing multiple data points to be evaluated when assessing a resident’s progression. A single checklist or global rating scale should not be the *only* measure defining SBR success. The ultimate success of any remediation plan should be improvement in the learner’s performance in the clinical environment.

Given the challenges of residency length constraints and learner variability in achieving competence, SBR can provide extra time and opportunities for struggling learners to train contemporaneously to routine simulations in order to achieve mastery within the CB framework. Although similar to non-remediation simulations in principle, what differentiates SBR from the former is the absolute need for confidentiality for ensuring a psychologically safe learning environment, low resident to faculty ratios, and the need for absolute transparency between the learner and the program leadership regarding the process (goals, objectives, results) of SBR. Also included in the latter is clear delineation of assessment methods and how their results will be used, especially as pertaining to high-stakes decisions such as “promotion” or “probationary” status. Unlike routine simulations, where to preserve psychological safety and safe-space principles, learner performance is not discussed outside of the debriefing room,^32^,^33^ in SBR learner performance is frequently discussed with residency leadership; the learners should be informed of this significant difference a priori.

## LIMITATIONS

The main limitations of this study are that it only represents EM residency programs from the United States. Caution should be used in applying these results to nursing and medical students and to other specialties and geographical locations. Although we met the stated guidelines for the size of the Delphi panel,^52^ the panel may have missed experienced simulationists. Our expert selection was dependent on available publications and presentations on SBR, of which there is a significant dearth. Although some of our experts have published or presented on this topic, most were identified through their actual experience in SBR, which in itself may not make them “experts” per se. Given the obvious lack of data on the subject, this approach seems reasonable. Additionally, although our survey instruments were developed using an iterative process, the length of the initial instrument could have contributed to survey fatigue and potential bias. Finally, a lack of face-to-face discussions to resolve disagreements may have limited some of our findings.

## CONCLUSION

This Delphi-based study, based on input from 30 ACGME-accredited EM programs across the United States, found agreement on many aspects of SBR. Simulation can be used diagnostically as well as therapeutically in remediation processes. Once a deficit is identified, simulation can be a helpful remediation tool for certain competencies and milestones, but simulation is not a one-size-fits-all approach that can be applied to every EM skill or competency. Simulation is best suited for remediation of procedural, patient care and communication milestones and less suited for remediation of systems-based practice and problem-based learning milestones. SBR can be one aspect, but should not be the sole component of a remediation plan. Similarly, SBR performance should only be one component of how remediation success is assessed by program leadership and the CCC, using tools with validity evidence when possible. These SBR assessments should be transparent between the simulation faculty, the learner, the program leadership and the CCC.

## Figures and Tables

**Table 1:** Emergency medicine residency programs represented in the Delphi panel.

Zucker School of Medicine-Hofstra/Northwell, New York Zucker School of Medicine-Hofstra/Northwell-Staten Island University Hospital, New York Yale New Haven Medical Center, Connecticut Icahn School of Medicine at Mt. Sinai, New York University of Connecticut, Connecticut Stanford University/Kaiser Permanente Medical Center, California Washington University/B-JH/SLCH Consortium, Missouri St. John’s Riverside Hospital, New York SUNY Health Science Center-Brooklyn, New York University of Missouri-Columbia, Missouri Icahn School of Medicine at Mount Sinai/St Luke’s-Roosevelt, New York Brown University, Rhode Island University of California-Davis, California Cook County Health and Hospitals Systems, Illinois University of Chicago, Illinois University of Florida College of Medicine-Jacksonville, Florida University of Arizona, College of Medicine-Tucson, Arizona University of California (UCLA) David Geffen School of Medicine/UCLA Medical Center/Olive View, California University of Illinois College of Medicine at Peoria, Illinois McGaw Medical Center of Northwestern University, Illinois University of Texas Southwestern, Texas Maimonides Medical Center, New York Boston University Medical Center, Massachusetts Virginia Commonwealth University, Virginia Indiana University School of Medicine, Indiana New York Presbyterian-University Hospitals of Columbia and Cornell University, New York Hennepin County Medical Center, Minnesota University of Pennsylvania, Pennsylvania New York Presbyterian Queens, New York Lehigh Valley Health Network, Pennsylvania

**Table 2:** Simulation-based remediation consensus statements.

Agreement strength*	Item
The role of simulation in remediation	
Strong agreement	Simulation can play a role in emergency medicine resident remediation.
	Simulation can be used as a diagnostic strategy for identifying specific learning deficits that may require remediation.
	Simulation can be used as a therapeutic strategy for addressing specific learning deficits.
	Simulation-based remediation (SBR) should be flexible with respect to topics and competencies to accommodate a wide variety of learner deficits.
	Various simulation modalities can be used to accommodate a variety of learner deficits. (For example, oral board-style tabletop simulations for medical knowledge remediation/clinical reasoning, partial task training for procedural remediation, high fidelity mannequin, and standardized patient simulation for communication/teamwork/situation awareness remediation/medical knowledge application/clinical skills).
The decision to use simulation for remediation	
Strong agreement	National organizations have recommended using simulations for teaching specific deficiencies and competencies; therefore, simulation can also be used to remediate the same deficiencies and competencies.
	SBR should be suggested by faculty or program leadership after learner assessments identify specific problems. (For example, specific learner deficits are realized at monthly evaluations or end of shift evaluations and discussed at faculty meeting or clinical competency committee [CCC] meetings or poor patient outcome).
	Learners should be informed of need for SBR by program leadership.
	SBR should be a part of a larger remediation process or plan.
	SBR should be conducted transparently such that the process of and performance during SBR are transparent not only to the learner, but also to the residency leadership and faculty involved in the resident’s remediation (i.e., CCC).
	The number of sessions and duration of SBR should be dependent on the issue being remediated and the resident’s performance and progress during each session.
Moderate agreement	SBR may be conducted by the program director/assistant program director (those ultimately involved in making progression decisions), as long as they have training in simulation.
	It is possible for procedure-based SBR to occur with only one session if competence is demonstrated at the end of the session.
The simulation-based remediation process	
Strong agreement	Ideally, SBR should be conducted by faculty who have formal simulation training/experience.
	SBR should occur one on one with the learner, unless the remediation concerns center around teamwork.
	If available, SBR cases should be pulled from a pool of cases with some validity evidence, provided the case objectives and goals apply to the specific situation (need/deficit) being remediated.
	If necessary, scenarios for SBR can be created de novo or pre-existing cases modified to address specific learner deficits or needs.
	SBR scenarios should be developed by faculty with simulation training and experience.
	Ideally, SBR should occur through multiple sessions.
Moderate disagreement**	The format of SBR should follow a standardized template or protocol.
Debriefing simulation-based remediation	
Strong agreement	SBR scenarios should always be followed by learner debriefing.
	The ideal debriefing method for SBR depends on the specific learner and the specific learning need and can be variable.
	The ideal debriefing method for SBR should be a blended approach such as PEARLS framework, which can include multiple debriefing modalities such as plus-delta and advocacy-inquiry.
Strong disagreement	The ideal debriefing method for SBR is blind debriefing by a third-party faculty based on a checklist/rubric filled out by simulation faculty.
Assessing and reporting simulation-based remediation	
Strong agreement	The format of SBR should be fluid and tailored to learner need or a specific deficiency identified.
	If available, learner assessment should be guided by checklists or rubrics with some validity evidence.
	Learner assessment may be guided by general critical action checklists that need not be “validated” but generally accepted per specialty guidelines.
	The length of SBR debriefing sessions can vary depending on the length of the simulation case, session objectives, and learner needs.
	If SBR occurs in a group setting with multiple learners, then the confidentiality of the learner requiring remediation must be maintained from other learners.
	SBR cases should be assessed formatively.
	Summative assessment may have a role in SBR, provided the cases have been specifically designed for it.
	If summative assessment is being used for SBR, learners should be informed ahead of time.
Strong disagreement	No report should be generated after SBR sessions, as this violates the “safe space” requirement for successful simulations.
Moderate disagreement	In SBR, learner assessment should be strictly scored per validated checklists or rubrics.
	SBR sessions should be confidential between the SBR faculty and the learner, and any report that is generated should remain confidential between the learner and SBR faculty.
	If a report is generated at the end of an SBR session, it should include definite statements like “credentialed” or “safe for independent practice.”
Defining and determining simulation-based remediation success	
Strong agreement	The definition of SBR success for a specific deficit must be clear, objective, measurable, and transparent.
	The definition of SBR success for a specific deficit must be set a priori, in collaboration with the learner, simulation faculty, and residency leadership collaboratively.
	Although checklists and global rating scales are a part of SBR assessment, they do not exclusively define SBR success, as they are focused on the simulation component and not the debriefing (where majority of learning occurs).
	One component of SBR success includes the learner developing insight into or awareness of his or her particular deficiencies as gauged through debriefing.
	Initial unsuccessful attempts at procedural SBR should require repeating the simulation session and successfully demonstrating that procedure.
	Initial unsuccessful attempts at non-procedure-based SBR should require completing another simulation session and successfully managing a different case with the same learning objectives.
	SBR success is defined by the learner appropriately addressing deficiencies in real-time clinical practice post simulation, as gauged by supervising clinical faculty. (For example, learner is demonstrating improved multi-tasking and patient dispositions in real time after sessions of SBR).
Moderate agreement	When SBR is being used as a diagnostic strategy to better identify/clarify learner deficits that require remediation, the ability of the faculty to identify or clarify one or more of these issues is what defines success.
Moderate disagreement	Successful SBR is defined exclusively by minimum passing scores on a critical action checklist and/or specific ratings on a global rating scale.
Deficiencies best addressed by simulation-based remediation	
Strong agreement	Application of medical knowledge
	Decision-making
	Clinical reasoning for high-acuity cases
	Procedural competencies
	Communication
	Teamwork
	Team-based resuscitations such as trauma/cardiac/pediatric codes
	Leadership in resuscitations
	Crisis resource management
	Multitasking (managing multiple patients simultaneously)
	Cognitive overload management for high-acuity cases
Moderate disagreement	Foundational medical knowledge
Sub-competencies best addressed by simulation-based remediation	
Strong agreement	1. Emergency Stabilization (patient care [PC]1) Prioritizes critical initial stabilization action and mobilizes hospital support services in the resuscitation of a critically ill or injured patient and reassesses after stabilizing intervention.
	2. Performance of Focused History and Physical Exam (PC2) Abstracts current findings in a patient with multiple chronic medical problems and, when appropriate, compares with a prior medical record and identifies significant differences between the current presentation and past presentations.
	4. Diagnosis (PC4) Based on all of the available data, narrows and prioritizes the list of weighted differential diagnoses to determine appropriate management.
	5. Pharmacotherapy (PC5) Selects and prescribes appropriate pharmaceutical agents based upon relevant considerations such as mechanism of action, intended effect, financial considerations, possible adverse effects, patient preferences, allergies, potential drug-food and drug-drug interactions, institutional policies, and clinical guidelines. Effectively combines agents and monitors and intervenes in the advent of adverse effects in the emergency department (ED).
	8. Multi-tasking (Task-switching) (PC8) Employs task switching in an efficient and timely manner in order to manage the ED.
	9. General Approach to Procedures (PC9) Performs the indicated procedure on all appropriate patients (including those who are uncooperative, at the extremes of age, hemodynamically unstable and those who have multiple comorbidities, poorly defined anatomy, high risk for pain or procedural complications, and sedation requirement), takes steps to avoid potential complications, and recognizes the outcome and/or complications resulting from the procedure.
	10. Airway Management (PC10) Performs airway management on all appropriate patients (including those who are uncooperative, at the extremes of age, hemodynamically unstable and those who have multiple comorbidities, poorly defined anatomy, high risk for pain or procedural complications, and sedation requirement), takes steps to avoid potential complications, and recognize the outcome and/or complications resulting from the procedure.
	14. Other Diagnostic and Therapeutic Procedures: Vascular Access. Successfully obtains vascular access in patients of all ages regardless of the clinical situation.
	22. Patient-centered Communication (ICS1) Demonstrates interpersonal and communication skills that result in the effective exchange of information and collaboration with patients and their families.
	23. Team Management (ICS2) Leads patient-centered care teams, ensuring effective communication and mutual respect among members of the team.
Moderate disagreement	17. Systems-based Management (SBP2) Participates in strategies to improve healthcare delivery and flow. Demonstrates an awareness of and responsiveness to the larger context and system of healthcare.
	19. Practice-based Performance Improvement (PBLI) Participates in performance improvement to optimize ED function, self-learning, and patient care.

* Strong agreement refers to free marginal kappa > 0.6 in the first round or total percent agreement, agreement >80% in the second round of the Delphi. Moderate agreement is defined as total percentage >70% in the second round.

** Strong disagreements refers to statements where total disagreement percent > 80% for strong and 70% for moderate levels of disagreement in first and second rounds of Delphi panel.

*PEARLS*, Promoting Excellence and Reflective Learning in Simulation debriefing approach.
